# Postpartum meloxicam administration alters plasma haptoglobin, polyunsaturated fatty acid, and oxylipid concentrations in postpartum ewes

**DOI:** 10.1186/s40104-020-00473-y

**Published:** 2020-07-01

**Authors:** Katie E. Olagaray, Barry J. Bradford, Lorraine M. Sordillo, Jeffery C. Gandy, Laman K. Mamedova, Turner H. Swartz, Trey D. Jackson, Emma K. Persoon, Caitlin S. Shugart, Curtis R. Youngs

**Affiliations:** 1 Department of Animal Sciences and Industry, Kansas State University, Manhattan, 66506 USA; 2 College of Veterinary Medicine, Michigan State University, 2265K Anthony Hall, East Lansing, MI 48824-1225 USA; 3 Department of Animal Science, Iowa State University, Ames, IA USA

**Keywords:** Eicosanoid, Inflammation, Lactation, Nonsteroidal anti-inflammatory drug, Sheep

## Abstract

**Background:**

Postpartum inflammation is a natural and necessary response; however, a dysfunctional inflammatory response can be detrimental to animal productivity. The objective of this study was to determine the effects of a non-steroidal anti-inflammatory drug (meloxicam) on ewe postpartum inflammatory response, ewe plasma polyunsaturated fatty acid and oxylipid concentrations, and lamb growth.

**Results:**

After lambing, 36 Hampshire and Hampshire × Suffolk ewes were sequentially assigned within type of birth to control (*n* = 17) or meloxicam orally administered on d 1 and 4 of lactation (MEL; 90 mg, *n* = 19). Milk and blood samples were collected on d 1 (prior to treatment) and d 4. Milk glucose-6-phosphate was not affected by MEL. Plasma haptoglobin (Hp) concentrations were less for MEL ewes; control ewes with greater d 1 Hp concentrations had elevated Hp on d 4, but this was not the case for MEL-treated ewes. Treatment with MEL increased plasma arachidonic acid concentration by more than 4-fold in ewes rearing singles but decreased concentrations of 9,10-dihydroxyoctadecenoic acid, prostaglandin F_2α_, 8-iso-prostaglandin E_2_, and 8,9-dihydroxyeicosatetraenoic acid. Nine oxylipids in plasma had interactions of treatment with d 1 Hp concentration, all of which revealed positive associations between d 1 Hp and d 4 oxylipid concentrations for CON, but neutral or negative relationships for MEL. MEL decreased 13-hydroxyoctadecadienoic acid:13-oxooctadecadienoic acid ratio and tended to increase 9-hydroxyoctadecadienoic acid:9-oxooctadecadienoic acid ratio (both dependent on d 1 values), indicating progressive metabolism of linoleic acid-derived oxylipids occurred by enzymatic oxidation after MEL treatment. Meloxicam reduced oxylipids generated across oxygenation pathways, potentially due to an improved redox state.

**Conclusions:**

Postpartum MEL treatment of ewes decreased plasma concentrations of Hp and several oxylipids, with the greatest impact in ewes with biomarkers reflecting a greater inflammatory state before treatment. Anti-inflammatory strategies may help resolve excessive postpartum inflammation in some dams.

## Background

Inflammation is a natural and necessary biological response to parturition; however, an uncontrolled inflammatory response can be detrimental to animal productivity. Postpartum inflammation has been well established in dairy cattle [1] with greater degrees of inflammation associated with decreased milk production [2], increased innate immune response [3], and decreased hazard of conception [4]. Use of nonsteroidal anti-inflammatory drugs (NSAID) to attenuate early lactation inflammation has been successful to increase both early lactation [5, 6] and whole-lactation milk production [7].

Increased plasma concentrations of the positive acute phase proteins α1-acid glycoprotein, haptoglobin, and ceruloplasmin, suggests sheep also experience postpartum inflammation [8]. To our knowledge, associations of postpartum inflammatory biomarkers with health and productivity of ewes and their lambs have not been evaluated, nor have there been any studies that investigated anti-inflammatory intervention strategies in sheep. Meloxicam is non-steroidal anti-inflammatory drug approved for use in Australia, New Zealand, and Canada, but the scope of research has been limited to its analgesic application during events such as lameness [9], castration, tail docking [10], and mulesing [11]. If postpartum meloxicam administration induces responses in ewes similar to those reported in dairy cattle [5–7], there is potential to improve ewe health and increase ewe milk production with subsequent increases in growth of suckling lambs.

Meloxicam decreases inflammation by inhibiting cyclooxygenase-2 (COX-2), the enzyme responsible for converting polyunsaturated fatty acids (PUFA) to oxylipids that include prostaglandins, thromboxanes, and leukotrienes [12]. The inhibitory action of meloxicam on COX-2 is known, but much remains to be elucidated with respect to the mechanism through which meloxicam changes physiology. The few plasma parameters that have been measured (Hp and serum amyloid A) in response to postpartum meloxicam treatment in cows were unaltered [7, 13, 14]. Despite the direct effect of meloxicam on one of the enzymatic pathways responsible for oxylipid synthesis, to our knowledge investigation of how meloxicam might create shifts within the oxylipid network has been limited to knee synovial fluid in horses [15] and humans [16].

Our primary objective was to determine if postpartum meloxicam administration to ewes would alter biomarkers of systemic inflammation. Our secondary objective was to determine the effect of postpartum meloxicam treatment of ewes on offspring growth. We hypothesized that postpartum meloxicam administration to ewes would reduce biomarkers of systemic inflammation, increase ewe milk production, and lead to greater lamb growth, particularly for ewes rearing twins.

## Methods

Experimental procedures were approved by the Iowa State University Institutional Animal Care and Use Committee (protocol #5-17-8532-O).

### Animals and treatments

Thirty-six ewes lambing during the 2018 winter lambing season at the Iowa State University Sheep Teaching Farm were used in a randomized design. At lambing ewes were sequentially assigned within type of birth (i.e. singleton, twin) to control (CON; *n* = 17) or treatment with meloxicam (MEL; *n* = 19). A dose of 90 mg of meloxicam, approximately 1 mg/kg BW (six 15 mg tablets in a #13 veterinary capsule; Torpac Inc., Fairfield, NJ) was administered orally within 24 h of lambing (d 1) and again on d 4 of lactation. Treatment time points were chosen for the first to be the most proximal time to lambing without hindering placenta expulsion [14], and the second to follow 3 d later, based on the 72 h clearance rate of meloxicam in sheep [17, 18].

Ewes and lambs were housed in a dry lot barn from the time of birth until weaning. Ewes typically lambed in communal lambing pens and were then moved into individual 4′ × 5′ postpartum acclimation pens for no more than 48 h. If a lamb was not thriving, the ewe and lamb(s) may have stayed in the individual postpartum pen for up to 96 h. The number of lambs born and reared were recorded. Fewer lambs were reared than born because of lamb death and removal of lamb(s) from ewes with insufficient milk. Descriptive statistics of ewes and lambs pre-treatment are presented in Table 1.

**Table 1:** Descriptive statistics for control ewes, ewes treated with 90 mg meloxicam on d 1 and 4 after lambing, and their lambs

	Control		Meloxicam	
	Mean ± SD	Range	Mean ± SD	Range
Ewe data				
*n*	17		19	
Breed				
Hampshire	12 (71%)		16 (84%)	
Hampshire × Suffolk	5 (29%)		3 (16%)	
Parity	4.2 ± 2.2	1–8	3.2 ± 2.1	1–7
Weight, kg	97.5 ± 15.1	73.5–122.0	90.4 ± 17.3	64.9–142.4
Total No. lambs born/trt	28		33	
Avg. No. lambs born/ewe	1.65 ± 0.49	1–2	1.74 ± 0.56	1–3
1	6 (33%)		6 (32%)	
2	11 (56%)		12 (63%)	
3	0		1 (5%)	
Avg. No. lambs reared	1.59 ± 0.51	1–2	1.63 ± 0.50	1–2
Total No. lambs reared	27		31	
1	7 (41%)		7 (37%)	
2	10 (59%)		12 (63%)	
Sire Breed				
Hampshire	13 (76%)		16 (84%)	
Hampshire × Suffolk	4 (24%)		3 (16%)	
Lamb data				
Sex of lamb				
Male	16 (59%)		16 (48%)	
Female	11 (41%)		15 (52%)	
Birth weight, kg	6.7 ± 0.7	5.2–8.4	6.4 ± 1.0	4.5–8.6

To avoid the potential stress associated with frequent sorting and weighing of lambs at specific d of age, which could adversely impact lamb growth performance, lambs were weighed and weaned in groups. Lambs were weighed at approximately 30 d of age (32 ± 2 d), weaning (61 ± 6 d of age), and approximately 30 and 60 d post-weaning (90 ± 5 and 120 ± 6 d of age, respectively). To reflect potential treatment effects on lamb weight gained per ewe, birth weights were removed, and lamb weights standardized to a constant d of age. For example, 30-d lamb weight gain was calculated by subtracting lamb birth weight from actual lamb weight (near 30 d of age) and then dividing the resultant value by the lamb’s age (in d) to acquire an average daily gain (ADG). The lamb’s ADG was then multiplied by 30 to achieve weight gained by 30 d. Because the effect of ewe meloxicam treatment on lamb growth was evaluated as lamb weight gained per ewe, for twin-rearing ewes it represents the combined weight gain of each twin lamb.

### Sampling and analysis

Blood samples were collected from each ewe within 24 h of lambing (immediately prior to MEL treatment) and again 3 d later. Samples were collected into 2 evacuated tubes (Thermo Fisher Scientific Inc. Waltham, MA), one containing heparin and the other K_3_EDTA, inverted several times, and placed on ice. Samples were centrifuged at 3000 × *g* for 15 min at 20 °C and plasma transferred to 1.5 mL microcentrifuge tubes for storage at − 80 °C until analyses. Prior to storing, plasma from EDTA tubes were snap frozen in liquid nitrogen for PUFA and oxylipid analyses. Milk samples were also collected from ewes on d 1 (to avoid colostrum) and d 4 of lactation. Milk samples were centrifuged at 1380 × *g* for 20 min at 4 °C. The fat layer was removed, and skim milk was stored at − 20 °C until analysis of glucose and glucose-6-phosphate (G6P).

Milk glucose and G6P concentrations were measured by a fluorometric assay as previously described [19, 20]. In short, G6P was determined through enzymatic oxidation by G6P dehydrogenase using NADP^+^ and the total (both glucose and G6P) was determined by enzymatic oxidation by both G6P dehydrogenase and hexokinase. Intra- and inter-assay coefficients of variation for G6P were 2.5% and 2.3%, and for glucose were 4.1% and 3.0%, respectively. Results are presented as both G6P concentration and G6P as a percent of total glucose available for phosphorylation.

Haptoglobin (Hp) was measured using a colorimetric assay that measures Hp-hemoglobin complexing via differences in peroxidase activity [21]. Haptoglobin concentrations from the colorimetric assay were validated using a commercial ELISA kit (cat#HAPT-11; Life Diagnostics Inc., West Chester, PA). Trolox equivalent antioxidant capacity (TEAC) was measured using a commercial antioxidant assay kit (#709001; Cayman Chemical; Ann Arbor, MI). Intra- and inter-assay coefficients of variation for haptoglobin were 3.5% and 4.3%, and for TEAC were 3.0% and 3.4%, respectively.

Plasma PUFA were analyzed with LC-MS and oxylipids by LC-MS/MS [22]. Briefly, 1 mL plasma was mixed with an antioxidant reducing agent mixture (50% methanol, 25% ethanol, and 25% water), butylhydroxytoluene (0.9 mmol/L), EDTA (0.54 mmol/L), triphenyphosphine (3.2 mmol/L), and indomethacin (5.6 mmol/L) to prevent ex vivo lipid peroxidation and oxidation of preformed oxylipids [23]. The following internal standards were added to each sample: 5(S)-hydroxyeicosatetraenoic acid-*d*_8_ (0.25 μmol/L), 15(S)-hydroxyeicosatetraenoic acid-*d*_8_ (0.25 μmol/L), 8,9-epoxyeicosatrienoic acid-*d*_11_ (0.5 μmol/L), prostaglandin E_2_-*d*_9_ (0.5 μmol/L), 8,9-dihydroxyeicosatrienoic acid-*d*_11_ (0.25 μmol/L), arachidonic acid-*d*_8_ (50 μmol/L), 2-arachidonoyl glycerol-*d*_8_ (2 μmol/L), and arachidonoyl ethanolamide-*d*_8_ (0.25 μmol/L) in 15 μL. A 7-point standard curve was generated with a mix of standards and internal standards for quantification.

Solid phase extraction was used for both PUFA and oxylipids [22]. Samples were reconstituted in a 2:1 methanol: HPLC-grade water mixture and passed through Acrodisc 13 mm GHP 0.2 μm syringe filters (Waters, Milford, MA) to remove any particulates. Samples were transferred to glass chromatography vials with glass inserts.

Fatty acids were quantified using a reverse phase LC on a Waters Acquity UPLC with a Supleco (State College, PA) Ascentis Express C18 10 cm × 2.1 mm, 2.7 μm column with a flow rate of 0.35 mL/min at 50 °C coupled to a quadrupole mass spectrometer. Mobile phases included A = acetonitrile, B = methanol, and D = 0.1% formic acid. The gradient mobile phase was programmed as follows (A/B/D ratio): time 0 to 0.2 min (45/22/33), to (80/19/1) at 4.0 min and maintained to 5.0 min, to (45/22/33) at 6 min and held until 8 min. Fatty acids were identified and quantified by matching mass-1 and retention time with corresponding internal standard and calibrated using a linear 7-point standard curve (R^2^ > 0.99).

Oxylipids were quantified using a Waters Acquity UPLC connected to a Waters Xevo-TQ-S tandem quadrupole mass spectrometer using multiple reaction monitoring. The Ascentis Express C18 HPLC column (Sigma Aldrich) was set at 50 °C and the autosampler at 10 °C. Flow rate was 0.3 mL/min. Eluents included 0.1% formic acid in water (A) and acetonitrile (B). The 15 min run time was programmed with a linear gradient as follows (A:B ratio): time 0 to 0.5 min (99:1), to (60:40) at 2.0 min, to (20:80) at 8.0 min, to (1:99) until 13.0 min, then returned to (99:1) at 13.01 min, and held until 15.0 min. Oxylipids were detected using electrospray ionization in negative-ion mode. Cone voltage and collision voltages were optimized for each analyte using Waters QuanOptimize software [23], and data analysis was carried out with Waters TargetLynx software.

All samples for fatty acid and oxylipids were analyzed in one batch. The signal to noise ratio was monitored; only data with a signal to noise ratio above 3 were considered detected and data with a signal to noise ratio ≥ 10 were used for calculations.

### Statistical analysis

Ewe plasma data (d 4) were analyzed using the MIXED procedure of SAS (version 9.4, SAS Institute, Cary, NC) with the fixed effects of treatment, d 1 covariate values, number of lambs born, and the two-way interactions of these variables, the quadratic term for d 1 covariate values and its interaction with treatment, the d 1 covariate for Hp, and the Hp covariate × treatment interaction. Ewe was included as a random effect. Treatment and covariates for outcomes of interest were retained in all models. Unless part of a significant interaction, all other variables were removed from the model when *P* > 0.20. Residual plots were assessed visually for normality. Any parameters violating that assumption were log-transformed prior to analysis with reported data back-transformed.

Weight of lamb raised per ewe at approximately 30, 60, 90, and 120 d of lamb age was analyzed using the MIXED procedure of SAS (version 9.4, SAS Institute, Cary, NC) with the fixed effects of treatment, number of lambs reared, lamb sex, and their two-way interactions, and with the random effect of ewe. Variables were removed from the model when *P* > 0.20.

All models utilized variance components as the covariance structure and removed observations when Studentized residuals were ≤ − 3 or ≥ 3. Significance was declared at *P* <  0.05 and tendencies at 0.05 ≤ *P* <  0.10.

## Results

### Ewe inflammatory, oxidative balance, and energy balance biomarkers

**Fig. 1:**
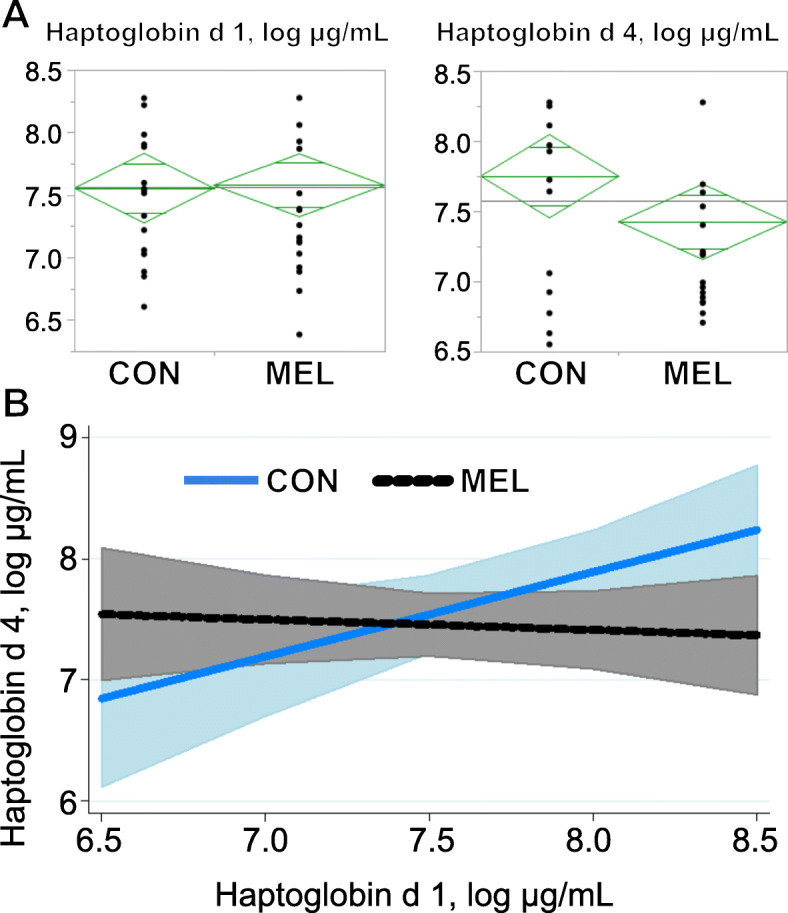
Baseline (d 1) haptoglobin relationship with d 4 haptoglobin is altered by meloxicam**. a** Log-transformed haptoglobin concentrations on d 1 (prior to treatment) and d 4 of lactation. The diamonds show means and 95% confidence intervals of the mean for each group. **b** Log haptoglobin concentration × treatment interaction (*P* = 0.04). Control ewes that had greater plasma haptoglobin concentration on d 1 had greater haptoglobin concentration on d 4, whereas initial plasma haptoglobin concentration on d 1 (before meloxicam) of treated ewes was not related to their d 4 values. D 4 haptoglobin concentration (log) = 8.10–0.09 × log haptoglobin covariate – 5.8[control] + 0.78 × log haptoglobin covariate[control]. Shaded areas represent 95% confidence intervals

Plasma Hp concentrations were lesser for MEL ewes, indicating reduced inflammatory status of ewes (*P* <  0.05); however, magnitude of the treatment response was dependent on d 1 Hp concentrations (*P* = 0.04; Fig. 1). As expected, there was a positive relationship between d 1 and d 4 Hp concentrations in control ewes, but meloxicam treatment eliminated this association. Ewes treated with MEL had similar d 4 Hp concentrations regardless of pre-treatment (d 1) concentration. Plasma TEAC, a measure of antioxidant capacity, did not differ between control and MEL ewes (*P* = 0.82). Milk G6P, an indirect indicator of ewe energy balance, was unaltered by MEL whether expressed as a concentration or as a percent of milk glucose (*P* ≥ 0.12; Table 2).

**Table 2:** Plasma biomarkers of inflammation (haptoglobin) and antioxidant capacity (TEAC) and milk markers of energy balance (G6P) in ewes treated with meloxicam at d 1 and 4 after lambing

				*P*-values^1^		
	CON	MEL	SEM	Trt	Cov	Cov × trt
Plasma						
Haptoglobin, μg/mL	2063	1713	275	< 0.05	NS	0.04
TEAC, mmol/L	1.00	1.00	0.02	NS	NS	NS
Milk						
G6P^2^, μmol/L	190.4	218.0	13.8	NS	0.01	0.09
G6P^3^, % of glucose	76.2	82.6	2.80	NS	NS	NS

^1^NS: *P* > 0.10; Cov: effect of d 1 covariate

^2^Cov × cov: *P* = 0.01; Cov × cov × trt: *P* = 0.08

^3^Effect of haptoglobin covariate: *P* = 0.05

### Ewe plasma polyunsaturated fatty acid concentrations

**Fig. 2:**
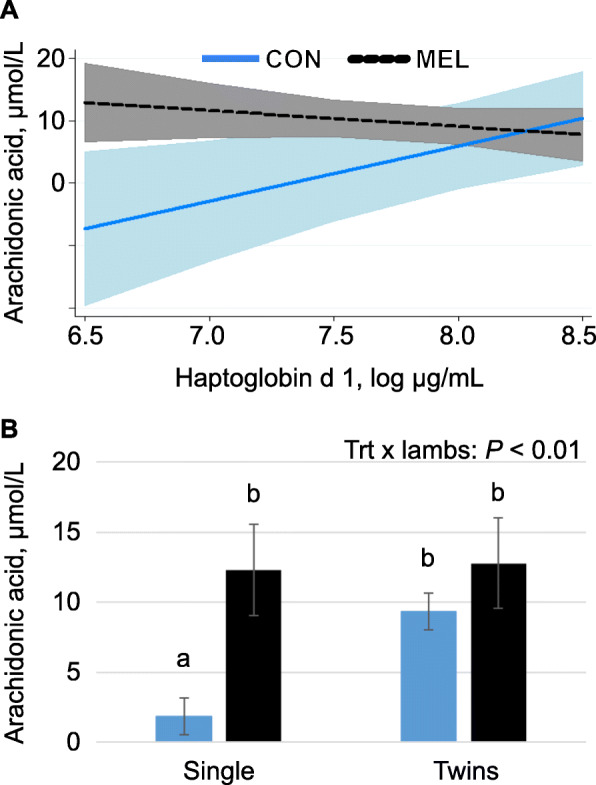
Meloxicam alters arachidonic acid relationships with baseline haptoglobin and type of rearing**. a** The treatment × haptoglobin covariate interaction (*P* = 0.01) for plasma arachidonic acid (ArA) concentration in control ewes (CON) and ewes treated with 90 mg meloxicam (MEL) on d 1 and 4 after lambing. D 4 ArA concentrations was dependent on d 1 haptoglobin concentrations (inflammation marker), with greater initial haptoglobin related to greater ArA in control ewes, but lesser ArA in ewes receiving MEL. D 4 ArA = 7.02–3.87 × covariate + 0.70 × covariate^2^–5.41[control] + 3.85 × covariate[control] – 0.77 × covariate^2^[control]. Shaded areas represent 95% confidence intervals; slopes differ at *P* = 0.01. **b** D 4 plasma ArA was less in control ewes rearing single lambs compared to MEL ewes rearing either single lambs or twins and control ewes rearing twins (Trt × lambs: *P* < 0.001)

Quantified plasma PUFA included linoleic acid (LA), arachidonic acid (ArA), eicosapentaenoic acid, dihomolinolenic acid, adrenic acid, and docosahexaenoic acid (Table 3). Among PUFA, ArA was the only one altered by MEL, with concentrations increased by more than 4-fold in ewes rearing singles (*P* <  0.01 main effect and interaction; Fig. 2).

**Table 3:** Plasma polyunsaturated fatty acid concentrations (μmol/L) in control ewes and ewes treated with 90 mg meloxicam on d 1 and 4 after lambing

					*P*-values^1^		
Fatty acid		CON	MEL	SEM	Trt	Cov	Lambs
Linoleic acid^2^	C18:2 (n-6)	97.61	86.50	16.07	NS	< 0.05	< 0.01
α-linolenic acid	C18:3 (n-3)	60.50	61.20	14.27	0.16	NS	< 0.01
Arachidonic acid^3^	C20:4 (n-6)	4.81	10.15	1.66	< 0.01	0.01	< 0.01
Eicosapentaenoic acid	C20:5 (n-3)	1.13	0.94	0.20	NS	NS	0.06
Dihomo-linolenic acid	C20:6 (n-6)	0.33	2.22	0.34	NS	0.03	NS
Adrenic acid	C22:4	0.016	0.013	0.002	NS	0.09	NS
Docosahexaenoic acid	C22:6 (n-3)	4.11	3.55	0.70	0.15	0.11	0.02
Total		263.6	289.6	40.4	NS	NS	< 0.01

^1^NS: *P* > 0.20; Cov: effect of d 1 covariate

^2^loghaptocov × trt: *P* = 0.03

^3^cov × trt: *P* = 0.01; cov × cov: *P* = 0.04; cov × cov × trt: *P* = 0.01; trt × lambs: *P* <  0.01; loghaptocov: *P* <  0.05; loghaptocov × trt: *P* <  0.01

### Ewe oxylipid profile

**Fig. 3:**
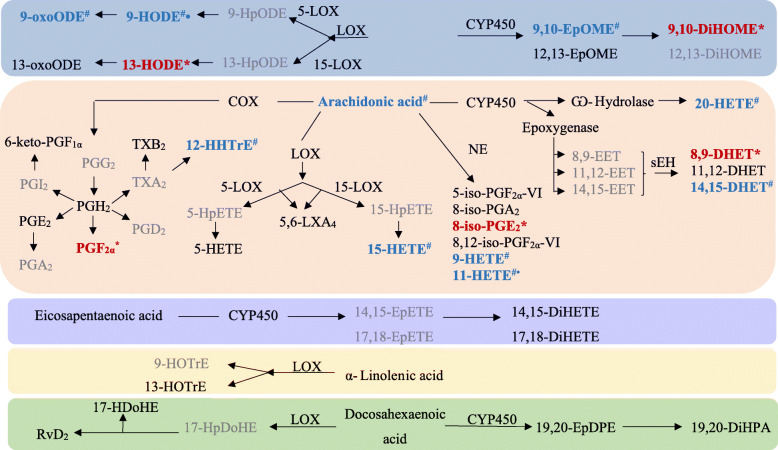
Oxylipid biosynthesis by fatty acid substrate and pathway. The effect of MEL administration to ewes on d 1 and 4 after lambing on oxylipid concentrations are shown with decreases represented in red text with an asterisk (*) and treatment × haptoglobin covariate represented in blue text and with a hashtag (#). Oxylipids that were not detected are in gray. The symbol (•) denotes oxylipids that can also be derived via non-enzymatic oxidation. Abbreviations: CYP = cytochrome P450; DHET = dihydroxyeicosatrienoic acid; DiHDoHE = dihydroxydocosahexaenoic acid; DiHDPA = dihydroxydocosapentaenoic acid; DiHETE = dihydroxyeicosatetraenoic acid; DiHOME = dihydroxyoctadecenoic acid; EET = epoxyeicosatrienoic acid; EpDPE = epoxydocosapentaenoic acid; EpOME = epoxyoctadecenoic acid; LX = lipoxin; HDoHE = hydroxyl-docosahexaenoic acid; HETE = hydroxyeicosatetraenoic acid; HHTrE = hydroxyheptadecatrienoic acid; HODE = hydroxyoctadecadienoic acid; HOTrE = hydroxyoctadecatrienoic acid; LOX = lipoxygenase; NE = nonenzymatic oxidation; oxoODE = oxooctadecadienoic acid; PG = prostaglandin; RvD_2_ = resolvin; sEH = soluble epoxide hydrolase; TXB_2_ = thromboxane B_2_

Plasma samples were analyzed for 57 oxylipids, 36 of which were detected and 31 of which were statistically analyzed. Although detectable, concentrations of PGD_2_, 11,12- epoxy-eicosatrienoic acid (EET), 14,15-EET, 6-keto-prostaglandin (PG) F_1α_, and 10,17- dihydroxydocosahexaenoic acid were very low and lacked sufficient variation to detect treatment differences. Effects of MEL on oxylipids are summarized by biosynthetic pathway (COX, lipoxygenase [LOX], cytochrome P450 [CYP], and non-enzymatic oxidation [NE]) in Tables 4, 5, 6, 7 and 8. Overall shifts to the oxylipid network can be visualized in Fig. 3.

**Table 4:** Cyclooxygenase-derived oxylipids in plasma in control ewes and ewes treated with 90 mg meloxicam on d 1 and 4 after lambing (mean ± SEM; μmol/L)

					*P*-values^1^			
Oxylipid^2^	Substrate^3^	CON	MEL	SEM	Trt	Cov	Cov × cov	Hpcov × trt
PGE_2_	ArA	0.19	0.15	0.03	NS	0.10	NS	NS
PGF_2α_	ArA	0.21	0.09	0.03	< 0.01	< 0.01	< 0.01	0.10
12-HHTrE	ArA	0.79	0.98	0.11	NS	NS	NS	0.04
TXB_2_	ArA	1.67	2.12	0.78	NS	NS	NS	NS

^1^NS: *P* > 0.10; Cov = d 1 covariate values

^2^*PG* Prostaglandin, *HHTrE* Hydroxyeicosatrienoic acid, *TXB*_*2*_ Thromboxane B_2_

^3^*ArA* Arachidonic acid

**Table 5:** Lipoxygenase-derived oxylipids in plasma of control ewes and ewes treated with 90 mg meloxicam on d 1 and 4 after lambing (mean ± SEM; μmol/L)

					*P*-values^1^				
Oxylipid^2^	Substrate^3^	CON	MEL	SEM	Trt	Cov	Cov × trt	Lambs	Hpcov × trt
5-HETE	ArA	0.10	0.29	0.22	NS	NS	NS	0.07	NS
15-HETE	ArA	2.03	1.96	0.16	NS	0.02	NS	NS	0.02
5,6-LXA_4_	ArA	0.13	0.16	0.03	NS	NS	NS	NS	NS
13(S)-HOTrE^4^	ALA	130.8	100.3	28.3	NS	0.08	0.08	NS	NS
17-HDoHE^5^	DHA	1.29	0.99	0.22	NS	< 0.01	NS	< 0.05	NS
RvD_2_	DHA	0.30	0.17	0.06	NS	NS	NS	NS	NS

^1^NS = *P* > 0.10; Cov = d 1 covariate values

^2^*HETE* Hydroxyeicosatetraenoic acid, *LXA*_*4*_ Lipoxin A_4,_*HOTrE* Hydroxyoctadecatrienoic acid, *HDoHE* Hydroxyl-docosahexaenoic acid, *RvD*_*2*_ Resolvin D_2_

^3^*ArA* Arachidonic acid, *ALA* α-Linolenic acid, *DHA* Docosahexaenoic acid

^4^Cov × cov: *P* = 0.08; cov × cov × trt: *P* = 0.08

^5^Cov × cov: *P* = 0.02; trt × lambs: *P* = 0.07

**Table 6:** Cytochrome P450-derived oxylipids in plasma in control ewes and ewes treated with 90 mg meloxicam on d 1 and 4 after lambing (mean ± SEM; μmol/L)

					*P*-values^1^					
Oxylipid^2^	Substrate^3^	CON	MEL	SEM	Trt	Cov	Cov × cov	Cov × trt	Lambs	Hpcov × trt
9,10-EpOME	LA	8.20	8.12	0.52	NS	< 0.01	NS	NS	NS	0.03
9,10-DiHOME	LA	22.13	17.55	1.05	< 0.01	< 0.10	NS	0.03	0.02	NS
12,13-EpOME	LA	24.30	24.09	1.68	NS	–	–	–	–	–
20-HETE	ArA	5.15	5.26	0.48	NS	NS	NS	NS	NS	0.04
8,9-DHET^4^	ArA	1.11	0.36	0.19	0.04	0.10	0.04	NS	0.04	0.07
11,12-DHET	ArA	1.48	1.48	0.13	NS	0.10	NS	NS	NS	NS
14,15-DHET	ArA	2.72	2.47	0.15	NS	< 0.001	< 0.01	NS	0.07	0.03
14,15-DiHETE	EPA	5.64	5.06	0.38	NS	< 0.01	< 0.01	0.10	0.07	NS
17,18-DiHETE	EPA	45.16	39.07	2.35	0.07	< 0.001	< 0.001	NS	< 0.01	NS
19,20-EpDPE	DHA	3.29	4.06	0.51	NS	NS	–	–	–	–
19,20-DiHDPA	DHA	2.31	2.47	2.39	NS	< 0.01	0.02	NS	< 0.01	0.09

^1^NS = *P* > 0.10; Cov = d 1 covariate values

^2^*EpOME* Epoxyoctadecenoic acid, *DiHOME* Dihydroxyoctadecenoic acid, *HETE* Hydroxyeicosatetraenoic acid, *DHET* Dihydroxyeicosatrienoic acid, *DiHETE* Dihyroxy-eicosatetraenoic acid, *EpDPE* Epoxydocosapentaenoic acid, *DiHDPA* Dihydroxydocosapentaenoic acid

^3^*LA* Linoleic acid, *ArA* Arachidonic acid, *EPA* Eicosapentaenoic acid, *DHA* Docosahexaenoic acid

^4^Cov × cov × trt: *P* = 0.05

**Table 7:** Nonenzymatic-derived oxylipids in plasma in control ewes and ewes treated with 90 mg meloxicam on d 1 and 4 after lambing (mean ± SEM; μmol/L)

					*P*-values^1^				
Oxylipid^2^	Substrate^3^	CON	MEL	SEM	Trt	Cov × trt	Lambs	Trt × lambs	Hpcov × trt
5-iso-PGF_2α_-VI	ArA	0.39	0.45	0.04	NS	–	–	–	–
8-iso-PGA_2_	ArA	0.40	0.45	0.06	NS	NS	NS	0.07	NS
8-iso-PGE_2_^4^	ArA	0.70	0.16	0.12	< 0.01	NS	< 0.01	0.08	NS
8,12-iso-PGF_2α_-VI	ArA	0.40	0.42	0.04	NS	NS	NS	NS	NS
9-HETE^5^	ArA	0.06	0.11	0.04	NS	0.04	NS	NS	0.03
11-HETE	ArA	1.07	1.03	0.12	NS	NS	NS	NS	< 0.01

^1^NS = *P* > 0.10

^2^*PG* Prostaglandin, *HETE* Hydroxyeicosatetraenoic acid

^3^*ArA* Arachidonic acid

^4^Haptocov: *P* = 0.06

^5^Cov × cov: *P* = 0.06

**Table 8:** Plasma concentrations of oxidized linoleic acid metabolites derived from multiple sources and ratios of select upstream:downstream metabolites in control ewes and ewes treated with 90 mg meloxicam on d 1 and 4 after lambing (mean ± SEM; μmol/L)

				*P*-values^1^				
Oxylipid or Ratio^2^	CON	MEL	SEM	Trt	Cov	Cov × trt	Lambs	Hpcov × trt
9-HODE	52.5	44.9	4.2	NS	0.01	0.07	0.03	< 0.01
9-oxoODE	13.99	12.37	1.24	NS	NS	NS	0.01	0.02
13-HODE	133.0	124.8	8.45	NS	NS	< 0.01	NS	NS
13-oxoODE	1.90	2.55	0.31	NS	NS	NS	NS	NS
9-HODE:9-oxoODE^3^	3.35	3.59	0.17	NS	NS	0.06	0.06	< 0.10
13-HODE:13-oxoODE^4^	62.3	51.1	5.4	NS	NS	0.04	NS	NS
9,10-EpOME:9,10-DiHOME	0.37	0.46	0.03	0.06	–	–	–	–

^1^NS = *P* > 0.10

^2^*HODE* Hydroxyoctadecadienoic acid, *oxoODE* Oxooctadecadienoic acid, *EpOME* Epoxyoctadecenoic acid, *DiHOME* Dihydroxyoctadecenoic acid

^3^Cov × cov × trt: *P* = 0.06

^4^Cov × cov: *P* = 0.09; Cov × cov × trt: *P* = 0.04

**Fig. 4:**
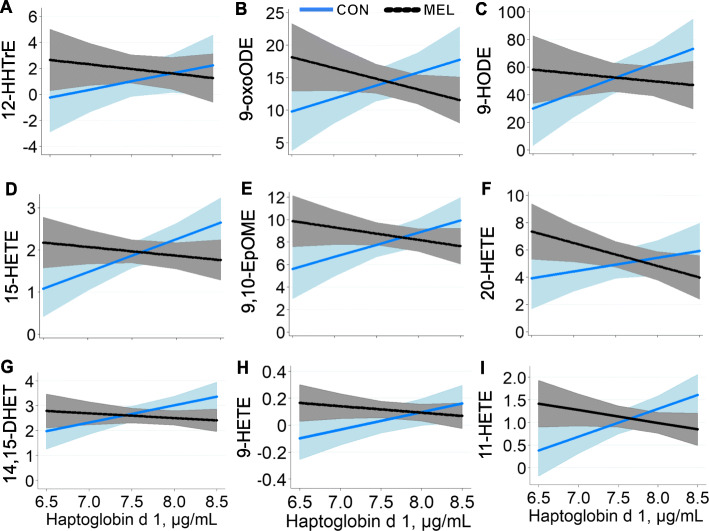
Meloxicam alters plasma oxylipid relationships with baseline haptoglobin. Treatment (trt) × haptoglobin covariate (log; loghaptocov) interactions for oxylipid concentrations (μmol/L) in control ewes (CON; blue line) and ewes treated with 90 mg meloxicam (MEL; black line) on d 1 and 4 after lambing. All interactions *P* < 0.05; shaded areas represent 95% confidence intervals. Cyclooxygenase-derived oxylipid: **a** 12-HHTrE. Lipoxygenase-derived oxylipids: **b** 9-oxoODE, **c** 9-HODE, **d** 15-HETE. Cytochrome P450-derived oxylipids: **e** 9,10-EpOME, **f** 20-HETE, **g** 14,15-DHET. Nonenzymatically derived oxylipids: **h** 9-HETE, **i** 11-HETE

The interaction between treatment and initial Hp concentration (Fig. 4) was significant or tended to be significant for many oxylipids (12- hydroxyeicosatrienoic acid [HHTrE], 11-hydroxyeicosatetraenoic acid [HETE], 9-hydroxyoctadecadienoic acid [HODE], 9-oxooctadecadienoic acid [oxoODE], 15-HETE, 9,10-epoxyoctadecenoic acid [EpOME], 20-HETE, 14,15-dihydroxyeicosatrienoic acid [DHET], and 9-HETE: *P* <  0.05; 8,9-DHET and 19,20- dihydroxydocosapentaenoic acid [DiHDPA]: *P* <  0.10). Generally, the observed interactions indicated a positive association between initial inflammatory status and the oxylipid in control ewes, but a negative relationship for MEL ewes.

The detected COX-derived oxylipids were all metabolites of ArA (Table 4). The only main effect of MEL on COX-derived oxylipids was decreased PGF_2α_.

The LOX-derived oxylipid concentrations are shown in Table 5. The α-linolenic acid metabolite 13-hydroxyocctadecatrienoic acid (HOTrE) tended to have a covariate × treatment interaction, with greater d 1 values related to decreased concentrations on d 4 for control, but little difference for MEL (*P* = 0.08). Ewes rearing twins had greater concentrations of 9-HODE (39.9 vs. 57.4 ± 4.8 μmol/L; *P* = 0.03), 9-oxoODE (10.7 vs. 16.2 ± 1.3 μmol/L), and 5-HETE (− 0.12 vs. 0.51 ± 0.23 μmol/L) compared with ewes rearing singletons. Concentration of 17- hydroxyl-docosahexaenoic acid (HDoHE) tended to have a treatment × lamb interaction with MEL attenuating the drop in HDoHE otherwise seen in twin-bearing ewes (MEL: 1.03 vs. 0.96 ± 0.29 μmol/L, CON: 1.92 vs. 0.66 ± 0.33 μmol/L; *P* = 0.07).

Effects of MEL on CYP-derived oxylipids are show in Table 6. Treatment with MEL tended to decrease 17,18- dihyroxy-eicosatetraenoic acid (DiHETE; *P* = 0.07). Concentrations of 9,10-DiHOME decreased with MEL, with the largest effect in ewes with greater initial concentrations (*P* = 0.03). MEL also decreased the ArA metabolite 8,9-DHET, and the cov^2^ × treatment interaction showed MEL prevented the decrease in d 4 concentrations for ewes with greater initial concentrations of 8,9-DHET (*P* = 0.05). Ewes raising twins had greater concentrations of 9,10-dihydroxyoctadecenoic acid (DiHOME; 18.0 vs. 21.7 ± 1.1 μmol/L), 8,9-DHET (0.66 vs. 0.81 ± 0.18 μmol/L), 17,18-DiHETE (36.5 vs. 47.8 ± 2.5 μmol/L), and 19,20-DiHDPA (1.83 vs. 2.96 ± 0.24 μmol/L; all *P* ≤ 0.04), and a tendency for greater concentrations of 14,15-DHET and 14,15-DiHETE (2.34 vs. 2.85 ± 0.16 μmol/L and 4.78 vs. 5.92 ± 0.40 μmol/L, respectively; *P* = 0.07) compared to ewes rearing a single lamb.

MEL effects on oxylipids formed through NE oxidation were mostly dependent on number of offspring reared and initial inflammatory status (Table 7). Concentrations of 8-iso-PGE_2_ were similar amongst control ewes raising singles, control ewes raising twins, and MEL ewes raising twins (0.51, 0.97, and 0.48 ± 1.5 μmol/L, respectively), but lesser for MEL ewes raising singles (0.06 ± 1.4 μmol/L). Control ewes with twins had the least 8-iso-PGA_2_ concentration (0.28 ± 0.07 μmol/L), control and MEL ewes with singles were intermediate (0.49 and 0.41 ± 0.09 μmol/L), and MEL ewes with twins were the greatest (0.52 ± 0.07 μmol/L). Treatment effects on 9-HETE were influenced by covariate values, in which 9-HETE decreased from initial values for control, but remained greater at d 4 for those treated with MEL (*P* = 0.04; Fig. 4h).

The oxidized LA metabolites 9- and 13-HODE can be formed from COX, LOX, CYP, or non-enzymatic (prooxidant) pathways; however, because racemic structures of these metabolites were not analyzed, we could not determine the relative proportion produced by each oxidation pathway. Treatment effects on 13-HODE and 9-HODE depended or tended to depend on d 1 covariate values (*P* <  0.01 and *P* = 0.07). Greater 13-HODE concentrations were positively related to d 4 concentrations for control, but negatively associated for MEL. Concentrations of 9-HODE were greater for MEL, and again there was a positive relationship between d 1 and 4 values in control ewes; however, post-treatment concentrations in MEL were independent of d 1 covariate values (cov × trt: *P* <  0.01).

**Fig. 5:**
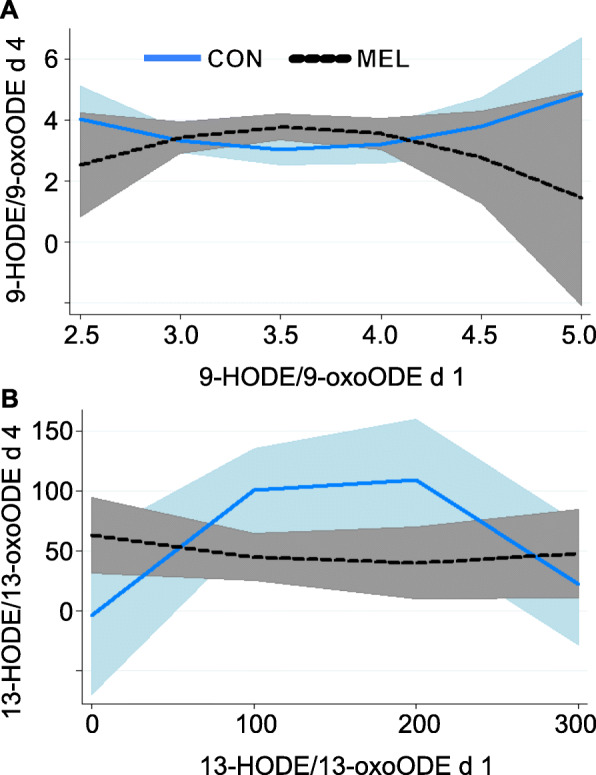
Meloxicam alters baseline haptoglobin associations with progressive metabolism of oxylipids**.** Linoleic acid-derived oxylipids in plasma for control ewes and ewes treated with 90 mg meloxicam on d 1 and 4 after lambing (mean ± SEM; μmol/L), expressed as ratios of select upstream:downstream metabolites. Shaded areas represent 95% confidence intervals and treatment (trt) is equal to 0 for MEL in the following eqs. **a** The 9-HODE/9-oxoODE ratio tended to have a treatment × covariate interaction (*P* = 0.06). Ewes with relatively greater proportion of 9-HODE at d 1 had a greater ratio on d 4 for MEL, but control ewes with a greater ratio at d 1 had a relatively lesser ratio at d 4. D 4 9-HODE/9-oxoODE = − 10.34 + 7.96 × covariate – 1.12 × covariate^2^ + 24.76[control] – 14.24 × covariate[control] + 2.00 × covariate^2^[control]. **b** The 13-HODE/13-oxoODE ratio tended to have treatment × covariate interaction (*P* = 0.06). Progressive metabolism from 13-HODE to 13-oxoODE was greater for MEL with greater initial ratio, but there was less progressive metabolism for CON ewes with greater initial ratios. D 4 13-Hode/13-oxoODE = 60.78–0.25 × covariate + 0.00065 × covariate^2^–66.97[control] + 1.77 × covariate[control] – 0.0054 × covariate^2^[control]

Differences in the progressive metabolism of oxylipids within certain pathways were assessed through ratios of hydroxyl oxylipids to their ketone derivatives (HODE to oxoODE) and epoxides to vicinal diols (EpOME to DiHOME). Ratios of 9-HODE:9-oxoODE and 13-HODE:13-oxoODE were both greater than 1 for both control and MEL, which signifies greater abundance of the hydroxyl oxylipids relative to their ketone derivatives. Initial 9-HODE:9-oxoODE tended to be positively related to values on d 4 for MEL but negatively related for control (*P* = 0.06; Fig. 5a). This ratio also tended to be related to initial inflammatory status with greater initial Hp related to slight increases for MEL and decreases for control (*P* ≤ 0.10). In contrast, the 13-HODE:13oxoODE ratio was less with MEL, with initial values positively related to d 4 values in control but negatively related in MEL (*P* = 0.04; Fig. 5b). Progressive metabolism of 9,10-EpOME tended to be lesser for MEL (*P* = 0.06); however, unlike the HODE:oxoODE ratios, there was a greater proportion of the downstream metabolite 9,10-DiHOME (ratio < 1).

### Lamb growth

Lamb weight produced per ewe did not differ by treatment at 30, 60, 90, or 120 d of age (Table 9). Unsurprisingly, type of rearing affected lamb weight produced, with ewes rearing twins producing more lamb weight than singles for all time points (*P* <  0.001).

**Table 9:** Weight of lamb produced per ewe (kg) at approximately 30, 60, 90, and 120 d after lambing for control ewes and ewes treated with 90 mg meloxicam on d 1 and 4 after lambing. To obtain average weight of an individual lamb at any time point, divide stated value by 1.58 (the average number of lambs reared per ewe) and add 6.3 kg (average birth weight)

				*P*–values^1^	
Age, d	CON	MEL	SEM	Trt	TOR^2^
30	14.9	15.6	0.6	NS	< 0.001
60	35.3	36.1	1.3	NS	< 0.001
90	55.0	55.7	1.7	NS	< 0.001
120	72.1	75.9	2.2	NS	< 0.001

^1^NS = *P* > 0.10

^2^*TOR* Type of rearing (single, twin). TOR × trt was tested but not significant

## Discussion

Despite the necessity of inflammatory signaling at parturition, a dysfunctional inflammatory response in this period has been attributed to health disorders and decreased animal productivity [1, 24]. Several studies in dairy cattle have investigated NSAID administration during the periparturient period to attenuate inflammation, but to our knowledge this is the first study to apply this intervention strategy to sheep. Our 2-dose regimen of MEL on d 1 and 4 after lambing decreased plasma haptoglobin concentrations, and the reduction was most pronounced for ewes with greater plasma Hp concentrations prior to treatment.

Although meloxicam is a selective inhibitor of COX-2 [25], treatment strategies that target only one enzymatic oxygenation pathway typically also have unpredicted effects on the entire oxylipid network [26]. For a thorough understanding of MEL effects on plasma oxylipid concentrations, we examined differences at each of the following levels that control oxylipid biosynthesis: 1) substrate (PUFA) availability, 2) products of multiple oxygenation pathways, and 3) the degree to which intermediate metabolites were catabolized to their end products [27].

The only difference we observed for PUFA concentrations was a 4-fold increase in ArA for MEL ewes bearing singletons. Although PUFA can be oxidized by each pathway, some pathways have a substrate preference [26]. In the case of COX-2, ArA is preferentially oxidized. Given MEL’s inhibition of COX-2, it is seemingly logical for the substrate to accumulate; however, ArA oxidation is expected to shift to other pathways in this scenario [25]. Additionally, it is unclear why MEL treatment decreased, rather than increased, ArA concentration in ewes with greater initial Hp concentrations. Because this result is somewhat counterintuitive, additional studies are needed to replicate the finding and explore potential explanations.

Direct downstream effects of COX inhibition were observed through the overall reduction in PGF_2α_ and decreased HHTrE in MEL ewes with greater pretreatment inflammation. As all COX-derived oxylipids detected in this study were metabolites of ArA, the observed effect for HHTrE could be partially attributed to reduced substrate availability. Effects on PGF_2α_ but not other prostanoids (PGD_2_, PGE_2_, PGI_2_) generated from the same substrate (PGH) could be due to the timing of our sample relative to the stage of inflammation [28]. For example, PGE_2_ is elevated during the early stages of inflammation compared with PGD_2_ which is more prevalent during the final stages of the response [29].

Meloxicam effects extended to oxylipids derived from the other oxygenation pathways, with the effects largely related to alterations in redox state and inflammatory status. As isoprostanes are only produced when there is a significant shift in redox state [24], they are considered the gold standard biomarkers of oxidative stress [30]. Thus, the observed decrease in 8-iso-PGE_2_ is one indicator that MEL reduced oxidative stress.

Oxylipids formed by NE oxidation also serve as indicators of oxidative stress and include 9-HETE, 11-HETE, and 9-HODE. Both 9-HETE and 11-HETE were decreased in MEL ewes with greater degrees of initial inflammation, suggesting a possible improved redox status. As both are predominately derived by NE oxidation of ArA, reduced substrate availability could partially explain the reductions in 9- and 11-HETE. Kuhn et al. [31] reported a similar scenario in which milk concentration of 11-HETE was elevated in early lactation; however, the significant correlation with its substrate ArA in milk (r = 0.60) only partially explained the elevation in their study. The greater oxidative environment of the mammary gland also contributed. Since the LA-derived 9-HODE can be produced both enzymatically via LOX or through NE oxidation, it can also serve as a marker of oxidative status [31]. Similar to 9 and 11-HETE, 9-HODE concentration was less in MEL ewes with greater degrees of initial inflammation, but since LA concentration was not different with MEL, this result can be more confidently attributed to the presence of fewer oxidants. Such a claim would be even further supported had we measured racemic structures of 9-HODE and could show that it was the proportion formed by NE that was reduced by MEL.

Decreased concentrations of oxylipids that contribute to a prooxidant environment would also indicate improved oxidative status. 20-HETE is a CYP-derived oxylipid that is not only a prooxidant itself, but also indirectly exacerbates oxidative stress via stimulation of mitochondrial reactive oxygen species production and activation of NADPH oxidase enzymes [32, 33]. Meloxicam decreased 20-HETE concentration in ewes with greater initial inflammation, which suggests decreased presence of reactive metabolites and thus improved redox status.

The initial products from 15-LOX oxidation of ArA and LA (15-HPETE and 13-HPODE, respectively) are also highly reactive and greatly contribute to oxidative stress [34]. Prior to LC-MS quantification, 15-HPETE and 13-HPODE had to be reduced to their hydroxyl and hydroperoxy derivatives. Thus, values for 15-HETE and 13-HODE represent the combined concentrations of the hydroperoxides and their hydroxyl derivatives. Treatment with MEL decreased concentration of 15-HETE in ewes with greater initial inflammation. Although relative contributions of 15-HPETE and 15-HETE cannot be teased apart in our data, our observed decrease in 15-HETE could have been driven by reductions in 15-HPETE. This alteration would further support improved oxidative status through decreased reactive metabolite availability.

The progressive metabolism of LA-derived oxylipids through the LOX pathway was also shifted. The ratios of 13-HODE:13-oxoODE and 9-HODE:9-oxoODE indicated further metabolism of 13-HODE to 13-oxoODE and less oxidation of 9-HODE to 9-oxoODE for MEL ewes compared to control. The further progression to 13-oxoODE is favorable because of its anti-inflammatory properties as a PPAR gamma ligand [35].

Soluble epoxide hydrolase (sEH), the enzyme that catalyzes the further metabolism of EETs to DHETs and EpHOMEs to DiHOMEs, is upregulated by prooxidant status [36]. Evaluation of ratios between these oxylipids and their downstream metabolites can provide some insight into sEH activity. The tendency for MEL to increase 9,10-EpOME/9,10-DiHOME could be the result of decreased sEH activity because of improved redox status. As DiHOME are more toxic than EpOME [37], the greater ratio in MEL ewes is preferable. Ratios of metabolites from CYP oxidation, EETs to DHETs, could not be evaluated. Although detected in some samples, EET concentrations were not statistically analyzed due to low concentrations with little variability. MEL did decrease 8,9-DHET overall and decreased 12,15-DHET in ewes with greater initial inflammation. Again, these reductions could indicate decreased sEH activity, but without knowing concentrations of their substrate precursors, we cannot differentiate between MEL shifting whole pathway flux vs. progression of oxidation within a pathway.

Oxylipids largely regulate inflammation by influencing the development of oxidative stress [24]. Our data reveal improved oxidant status for ewes treated with MEL, especially for those with greater initial degrees of inflammation. The haptoglobin covariate × treatment interaction was significant for 9 oxylipids with an additional 2 tending to be significant. The interaction for each of these were in the same direction: decreased oxylipid concentrations for MEL ewes with greater inflammation that were otherwise increased for control ewes. The fact that characteristically pro- and anti-inflammatory oxylipids were both altered in a similar manner demonstrates the natural balance within the complex oxylipid network. Not only does it seem oxylipids balance each other, but individual oxylipids can have different effects based on the receptor present on target cells or stage of inflammation. For example, 8-iso-PGE_2_ promotes vasoconstriction when working through the thromboxane receptor, but vasodilation through the PGE_2_ prostanoid receptor [38]. Overall, our results demonstrate MEL decreased systemic inflammation in ewes with greater degrees of initial inflammation, in part because of alterations to oxylipid biosynthesis across multiple oxidation pathways.

The many interactions we observed with initial inflammatory status could be a possible explanation to the inconsistency of NSAID response in transition dairy cattle studies. For example, Carpenter et al. [7] reported substantial whole-lactation milk yield responses after oral administration of sodium salicylate to dairy cows for 3 d after calving; however, when the study was later replicated, no difference in milk production through 120 d was observed [39]. As plasma haptoglobin concentrations were nearly 3-fold greater in the former cohort (~ 600 vs. 200 μg/mL), authors speculated a milk response to NSAID treatment could be dependent on baseline inflammation [39]. As discussed throughout the paper, our data supports the notion that response to postpartum NSAID treatment in ruminants is dependent on initial inflammatory status.

Despite MEL decreasing inflammation, no treatment effect was observed for milk G6P, an indirect indicator of energy balance. Zachut and others [20] have demonstrated a negative linear correlation between milk G6P concentration and energy balance (r = − 0.45). As systemic inflammation is correlated with decreased feed intake, we hypothesized alleviation of inflammation would promote feed intake and greater energy balance. Feed intake and milk yield data were not available, therefore analysis of milk G6P was employed to gain some insight into energy balance. To our knowledge, this is the first experiment to report milk G6P concentrations in sheep milk; values for controls were only slightly lower than concentrations reported for dairy cattle on d 3 of lactation (200–350 μmol/L [40]).

Postpartum meloxicam treatment of ewes did not affect lamb weight produced per ewe (a proxy of lamb growth); however, our small sample size likely limited our ability to detect a statistical difference. It is also possible that a greater dose of MEL (e.g. 2 mg/kg BW) may have yielded differences in milk yield and growth of suckling lambs, although a recent study [9] could argue against the potential benefit of a greater dose. Future studies should reevaluate the hypothesis that postpartum MEL might increase ewe milk production and thereby increase lamb growth, especially in ewes bearing multiple offspring. Even though ewes suckling twin lambs produce 17–61% more milk than ewes suckling single lambs [41], the nutrient supply is split between the 2 lambs, resulting in 60–80% of the nutrients (20–40% less compared to a singleton). Increasing nutrient supply via increased milk production could increase pre-weaning lamb growth, thereby increasing profit potential for sheep producers.

## Conclusions

Postpartum meloxicam administration decreased ewe inflammatory status as measured by plasma haptoglobin, with reductions greatest for those with greater initial haptoglobin concentrations. MEL increased plasma ArA concentrations in ewes bearing singletons, but decreased ArA in ewes with greater initial inflammation. Meloxicam also decreased plasma concentrations of an array of oxylipids extending across different PUFA substrates and oxidation pathways, and altered their progressive metabolism. Many of the oxylipid MEL effects pointed to improved redox state that paralleled the reductions in inflammation. No differences in lamb growth were detected, but future research with a larger sample size, particularly of twins and triplets, is warranted.

## Data Availability

The datasets used and/or analyzed during this study are available from the corresponding author on reasonable request.

## Abbreviations

ArA: Arachidonic acid
COX: Cyclooxygenase
CYP: Cytochrome P450
DiHDoHE: Dihydroxydocosahexaenoic acid
DiHDPA: Dihydroxydocosapentaenoic acid
DiHETE: Dihydroxyeicosatetraenoic acid
DiHOME: Dihydroxyoctadecenoic acid
DHET: Dihydroxyeicosatrienoic acid
EET: Epoxy-eicosatrienoic acid
EpDPE: Epoxydocosapentaenoic acid
EpOME: Epoxyoctadecenoic acid
FA: Fatty acid
G6P: Glucose-6-phosphate
HDoHE: Hydroxyl-docosahexaenoic acid
HETE: Hydroxyeicosatetraenoic acid
HHTrE: Hydroxyeicosatrienoic acid
HODE: Hydroxyoctadecadienoic acid
HOTrE: Hydroxyocctadecatrienoic acid
Hp: Haptoglobin
LnA: Linoleic acid
LOX: Lipoxygenase
LXA_4_: Lipoxin A_4_
MEL: Meloxicam
NE: Nonenzymatic oxidation
OxoODE: Oxooctadecadienoic acid
PG: Prostaglandin
PUFA: Polyunsaturated fatty acid
RvD_2_: Resolvin
sEH: Soluble epoxide hydrolase
TEAC: Trolox equivalent antioxidant capacity
TXB_2_: Thromboxane B_2_
